# Prevalence of alcohol use before and during pregnancy and predictors of drinking during pregnancy: a cross sectional study in Sweden

**DOI:** 10.1186/1471-2458-13-780

**Published:** 2013-08-27

**Authors:** Janna Skagerström, Siw Alehagen, Elisabet Häggström-Nordin, Kristofer Årestedt, Per Nilsen

**Affiliations:** 1 Department of Medical and Health Sciences, Division of Community Medicine, Linköping University, Linköping, SE-581 83, Sweden; 2 Department of Medical and Health Sciences, Division of Nursing Science, Faculty of Health Sciences, Linköping, SE-581 85, Sweden; 3 Department of Women's and Children's Health, Uppsala University, Uppsala, Sweden; 4 School of Health, Care and Social Sciences, Mälardalen University, Västerås, Sweden; 5 Department of Medical and Health Sciences, Division of Health Care Analysis, Linköping University, Linköping, SE-581 83, Sweden

## Abstract

**Background:**

There is a paucity of research on predictors for drinking during pregnancy among women in Sweden and reported prevalence rates differ considerably between studies conducted at different antenatal care centres. Since this knowledge is relevant for preventive work the aim of this study was to investigate these issues using a multicenter approach.

**Methods:**

The study was conducted at 30 antenatal care centers across Sweden from November 2009 to December 2010. All women in pregnancy week 18 or more with a scheduled visit were asked to participate in the study. The questionnaire included questions on sociodemographic data, alcohol consumption prior to and during the pregnancy, tobacco use before and during pregnancy, and social support.

**Results:**

Questionnaires from 1594 women were included in the study. A majority, 84%, of the women reported alcohol consumption the year prior to pregnancy; about 14% were categorized as having hazardous consumption, here defined as a weekly consumption of > 9 standard drinks containing 12 grams of pure alcohol or drinking more than 4 standard drinks at the same occasion. Approximately 6% of the women consumed alcohol at least once after pregnancy recognition, of which 92% never drank more than 1 standard drink at a time. Of the women who were hazardous drinkers before pregnancy, 19% reduced their alcohol consumption when planning their pregnancy compared with 33% of the women with moderate alcohol consumption prior to pregnancy. Factors predicting alcohol consumption during pregnancy were older age, living in a large city, using tobacco during pregnancy, lower score for social support, stronger alcohol habit before pregnancy and higher score for social drinking motives.

**Conclusions:**

The prevalence of drinking during pregnancy is relatively low in Sweden. However, 84% of the women report drinking in the year preceding pregnancy and most of these women continue to drink until pregnancy recognition, which means that they might have consumed alcohol in early pregnancy. Six factors were found to predict alcohol consumption during pregnancy. These factors should be addressed in the work to prevent alcohol-exposed pregnancies.

## Background

The association between alcohol use and numerous adverse health consequences for the fetus and the developing child later on in life has been well documented since the 1970s [1,2]. Research on the effects of small amounts has shown differing results. In a review of published studies, Henderson and colleagues concluded that there is no consistent evidence of the harm caused by small to moderate amounts of alcohol consumed during pregnancy [3]. Further studies on specific outcomes published after the review support this conclusion [4-6]. However, no level of alcohol consumption has been determined as completely safe during pregnancy. Therefore, total abstinence during pregnancy is recommended by policy makers in many countries, including Sweden [7].

Sweden has experienced an increase in alcohol consumption during the last decades [8]. Simultaneously, there has been a progressive increase in the age of Swedish women giving birth to their first child, from 23.8 years in 1973 to 28.9 years in 2009 [9] and many women have consumed alcohol for 15 years or more when they begin their first pregnancy. This potentially makes it more difficult to cease drinking when becoming pregnant and abstain throughout the pregnancy.

Previous studies in Sweden have been conducted at single antenatal care centers in three large cities (Stockholm, Linköping, and Uppsala) and have reported prevalence rates of drinking during pregnancy ranging from 6% to 30% [10-12]. However, differences in methodology make it difficult to compare the results of these studies. Population-based studies show that alcohol consumption in Sweden varies between different regions of the country; consumption is higher in larger cities than in rural areas [8]. The extent to which there might be corresponding patterns of drinking during pregnancy has not previously been investigated in Sweden.

Predictors for drinking during pregnancy identified in previous Swedish studies include the Alcohol Use Disorder Identification Test (AUDIT) score for drinking prior to pregnancy, higher frequency of pre-pregnancy drinking, higher maternal age, having given birth before, and nicotine use in early pregnancy [10-12]. International studies have reported on the relevance of a wide range of potential predictors for drinking during pregnancy that have not been confirmed in a Swedish context, including education level, employment status, civil status, and social support [13-15]. Research on behavioral changes in various domains suggests that it might be important to explore the extent to which pre-pregnancy drinking constitutes a habit that inhibits the ability to cease drinking when becoming pregnant [16,17]. The aim was to investigate alcohol use before and during pregnancy and predictors for drinking during pregnancy in Sweden. This knowledge could potentially inform and improve preventive efforts in antenatal care.

## Methods

### Ethical approval

This study has been approved by the Regional Ethical Board in Linköping (Dnr M178-09).

### Study setting

The study was conducted at 30 antenatal care centers in Sweden from November 2009 to December 2010. Each participating center collected data from pregnant women during a 4-week period. Sweden has a comprehensive system of public antenatal care units, which reach nearly all pregnant women. In 2008, there were 122 701 women registered at 442 antenatal care centers in Sweden [18]. A strategic selection of antenatal care centers across Sweden was recruited for the study based on the distribution of pregnant women in 2008. The goal was to recruit centers that were representative of the distribution of pregnant women based on two dimensions: geographic location of the center (three regions in Sweden: Norrland, Svealand, and Götaland) and population size (major city, >200 000 inhabitants; medium-sized city, 50 000–200 000 inhabitants; or other city, <50 000 inhabitants or rural area).

### Data collection and study participants

Consecutive pregnant women who had reached the 18th week of their pregnancy with a scheduled midwife consultation at any of the 30 antenatal centers were asked to participate in the study. The questionnaire was in Swedish and no translation was provided why non Swedish-speaking women were excluded from the study. Each woman was informed that participation in the study was voluntary. The midwife at the end of the consultation gave an anonymous questionnaire and information about the purpose of the study to the women. The women were asked to fill out the questionnaire in the waiting room (where no midwife was present). When finished, the questionnaire was sealed in an envelope and put into a box. If the woman declined to participate, the midwife asked a few questions for a short drop-out questionnaire covering the woman’s age, whether she had previous children, pregnancy week, and number of antenatal care visits.

Of the 1693 women who were asked to participate, 1637 agreed. The most common reason given for nonparticipation was lack of time. A further 43 women were excluded from the analysis because the respondent had neglected to answer most questions, had provided contradictory answers, or had not answered the questions on alcohol (25 questionnaires). Of the remaining questionnaires, 20 did not have information on either quantity or frequency of alcohol consumption before pregnancy which made it impossible to categorize them as moderate or hazardous drinkers. For this reason, the analyses where the study population was stratified into drinking categories have been conducted on 1574 questionnaires and 1319 questionnaires when abstainers were excluded. For the logistic regression, the study population was 1594 minus 253, abstainers leaving 1341 questionnaires for the analysis. The internal dropout ranged between 0.7 and 5.1% on the variables included in the analyses.

Of the women asked to participate 56 women declined. The non-responders were younger than the participants (*p*<0.001) but did not differ regarding whether they already had children, pregnancy week, or number of visits in antenatal care.

### Questionnaire and study variables

The questionnaire consisted of 22 questions on alcohol consumption before and during pregnancy, drinking motives, consumption habits, tobacco use and social support. Further questions concerned background characteristics of the respondents.

Alcohol consumption prior to and during the pregnancy was measured using AUDIT-C consisting of the first three items of AUDIT measuring quantity and frequency of alcohol consumption and frequency of heavy episodic drinking [19]. The response options concerning alcohol consumption during pregnancy were slightly modified to be applicable for a pregnant population, for example 1-2 times during the whole pregnancy was added as an option. The questionnaire also included one yes/no item on whether the woman had reduced her alcohol consumption in association with her pregnancy. Women who answered “yes” were also asked about the time point when they reduced their consumption: “when I planned my pregnancy”; “when I became aware I was pregnant”, “after my first appointment with a midwife,” or “other”.

Strength of the prepregnancy alcohol habit was measured using a five-item version of the Self-Rated Habit Strength Index (SRHI) [20]. The five items are scored 1-4 with a maximum total score of 20, weaker habit yielding higher score. The SRHI was originally a 12-item instrument, but the adapted version was developed by the last author of this study in collaboration with Verplanken to achieve a more feasible instrument. The five-item version has previously been tested with a satisfactory internal consistency supporting its use. Several shorter versions of SRHI have been used with no apparent losses in reliability [21-23]. In the present study Cronbach’s alpha was 0.82

Prepregnancy drinking motives were measured with the Drinking Motives Questionnaire (DMQ) [24]. DMQ investigates three types of reasons for drinking: coping motives, social motives, and enhancement motives. Each drinking motive is investigated by five items answered on a four-point scale. In the present study Cronbach’s alpha for the different motives were: social motives 0.71, coping motives 0.78, and enhancement motives 0.76.

Tobacco use before and during pregnancy was investigated with two questions. Both questions addressed the two most commonly used tobacco products in Sweden: cigarettes and Swedish oral moist snuff. The question on tobacco use before pregnancy had four response options (using tobacco daily, using but not daily, have been using tobacco regularly but quit before pregnancy recognition, have never been a regular tobacco user). The question on tobacco use during pregnancy had three response options (have been using tobacco daily during pregnancy, have been using tobacco during pregnancy but not daily, have not used tobacco during pregnancy).

Perceived social support was assessed with the Maternity Social Support Scale (MSSS) [25]. The scale includes one question each on perceived support from friends and family, and four questions on perceived support from husband/partner. The scores for each item on MSSS can range between 5 and 30, higher support yielding higher score.

### Data analysis

For categorical variables the chi-square test and Fisher exact test, when appropriate, were used to compare nonresponders with responders, and hazardous drinkers, moderate drinkers and abstainers before pregnancy. Instruments generating a sum-score were compared using Mann–Whitney *U* test.

Occupation was collapsed into two categories: employed or other (including studying, unemployed, parental leave/on leave, sick leave, and other). Age was coded into five categories; ≤ 24, 25-29, 30-34, 35-39 and ≥ 40.

Three categories of alcohol consumption were constructed from the answers from the questions on alcohol consumption before pregnancy: abstainers were defined as the women who answered that they had not been drinking in the past 12 months; moderate drinkers had a weekly consumption of 1–9 standard drinks (SD) (one standard drink contains 12 grams of pure alcohol) and engaged in binge drinking less than once a month; and hazardous drinkers had a weekly consumption of >9 standard drinks or engaged in binge drinking once a month or more often. Binge drinking was defined as drinking more than four standard drinks on one occasion, five drinks equals about 60 g of pure alcohol, the cut-off proposed by the WHO [26]. This definition of hazardous drinking (also referred to as risk drinking) has been used by the Swedish National Institute of Public Health [27]. Two categories of alcohol consumption during pregnancy were constructed: abstainers during pregnancy were women who reported that they did not drink any alcohol at all after becoming aware of their pregnancy and drinkers during pregnancy were those who reported that they had consumed alcohol at least once after pregnancy recognition. Thus, any drinking during pregnancy, even very small amounts, was categorized as drinking during pregnancy.

Binary logistic regression analysis with a backward elimination procedure was carried out to investigate predictors for alcohol use during pregnancy. All women who used alcohol in the year preceding or during pregnancy were included in the regression model, whereas women who were abstainers before and during pregnancy were excluded. The dependent variable, any use, comprised the frequency question recoded into “never been drinking alcohol since pregnancy recognition” and “any drinking since pregnancy recognition”. The included independent variables were age, education, city size, region, quantity and frequency of drinking before pregnancy, frequency of binge drinking before pregnancy, tobacco use before and during pregnancy, social support, habits and drinking motives.

Dummy coding was used for all categorical predictor variables. No problem with multicollinearity between the predictor variables were detected according to the variance inflation factor (VIF, range= 1.04-1.85, mean= 1.36). The regression model demonstrated good model fit according to Hosmer and Lemeshow goodness-of-fit test (*χ*^2^(8)=5.63, *p*=0.689). Results are reported as odds ratios, with 95% confidence intervals and *p*-values. All results were considered significant at *p*≤ 0.05. PASW Statistics 18 software was used for the statistical calculations.

## Results

### Population characteristics

Two-thirds of the women were aged 25-34 years, 21% was 35 years of age or older and 12% was 24 or younger. More than half of the women had a university education. Four-fifths were employed. Almost all (95%) the respondents were married or cohabiting. The distribution of primiparous and multiparous women was similar.

### Alcohol use before pregnancy

Sixty-eight percent of the women reported moderate alcohol consumption in the year preceding the pregnancy. Prepregnancy abstinence was reported by 16%. Hazardous drinking due to weekly consumption (>9 standard drinks) was reported by 36 women (2.3%) and hazardous consumption due to binge drinking frequency (binge drinking once a month or more) was reported by 219 women (13.7%). Twenty-six women reported hazardous alcohol consumption for both dimensions (weekly consumption and binge drinking frequency), which meant that 229 (14.4%) women were categorized as having hazardous consumption for at least one dimension.

There were considerable differences between the three prepregnancy drinking categories with regard to age, education, occupation, civil status, city size, and region (Table 1). More moderate drinkers had a college or university education than both hazardous drinkers and abstainers. A smaller proportion of the abstainers were employed when becoming pregnant. Being married or cohabiting with a partner was more common among the moderate drinkers; hazardous drinkers were more often single. A larger proportion of the hazardous drinkers lived in a major city compared with both abstainers and moderate drinkers.

**Table 1:** Sociodemographics and characteristics of the participants

	**Alcohol intake in the year prior to the pregnancy**				
	**Total, *n *(%)**	**Abstainers, *n *(%)**	**Moderate drinkers, *n *(%)**	**Hazardous drinkers, *n *(%)**	***p*-value**
Age (years)	n=1563	n=253	n=1081	n=229	<0.001
≤ 24	184 (11.8)	45 (17.8)	95 (8.8)	44 (19.2)	
25–29	513 (32.8)	86 (34.0)	359 (33.2)	68 (29.7)	
30–34	535 (34.2)	61 (24.1)	390 (36.1)	84 (36.7)	
35–39	280 (17.9)	54 (21.3)	198 (18.3)	28 (12.2)	
≥ 40	51 (3.3)	7 (2.8)	39 (3.6)	5 (2.2)	
Education	n =1539	n =249	n =1068	n =222	<0.001
Compulsory school	50 (3.2)	25 (10.0)	17 (1.6)	8 (3.6)	
Intermediate education	621 (40.4)	130 (52.2)	384 (36.0)	107 (48.2)	
University/college education	868 (56.5)	94 (37.8)	667 (62.5)	107 (48.2)	
Occupation	n=1562	n=253	n=1081	n=227	<0.001
Employed	1250 (80.0)	172 (68.0)	896 (82.9)	182 (79.8)	
Other	312 (20.0)	81 (32.0)	185 (17.1)	46 (20.2)	
Civil status	n=1562	n=252	n=1081	n=229	<0.001
Married or cohabiting	1485 (95.0)	232 (92.1)	1048 (96.9)	205 (89.5)	
In a relationship (live apart)	44 (2.8)	14 (5.6)	20 (1.8)	10 (4.4)	
Single	34 (2.2)	6 (2.4)	14 (1.3)	14 (6.1)	
City size*	n=1574	n =255	n =1090	n =229	0.001
>500 000	503 (32.0)	72 (28.2)	334 (30.6)	97 (42.2)	
<500 000	1071 (68.0)	183 (71.8)	756 (69.4)	132 (57.6)	<0.001
Region**	n =1574	n =255	n =1090	n =229	
Norrland1)	519 (33.0)	82 (32.2)	374 (34.3)	63 (27.5)	
Svealand2)	336 (21.3)	27(10.6)	239 (21.9)	70 (30.6)	
Götaland3)	719 (45.7)	146 (57.3)	477 (43.8)	96 (41.9)	

*City size where the antenatal care center was located (not always where the women lived). **Regions where the antenatal care center was located. The regions referred to are easily described as the northern, middle, and southern parts of the country. The regions are traditional and each region consists of a number of provinces.

Hazardous drinkers scored lower on the habit index, meaning they had stronger prepregnancy alcohol habits compared with moderate drinkers. Also, it was more common among the hazardous drinkers to use tobacco during pregnancy. The moderate drinkers more often than the hazardous drinkers ceased drinking when planning their pregnancy (as opposed to stopping when becoming aware of their pregnancy), and used tobacco before pregnancy (Table 2).

**Table 2:** Strength of habit, social support, time point at which drinking reduced tobacco use and drinking motives among moderate and hazardous drinkers

	**Moderate drinkers, *n *(%)**	**Hazardous drinkers, *n *(%)**	***p*-value**
Strength of habit (lower score=stronger habit)	n=1068	n=223	<0.001^a^
4-10 points	10 (0.9)	22 (9.9)	
11-15 points	87 (8.1)	65 (29.1)	
16-20 points	971 (90.9)	136 (61.0)	
Time point when alcohol reduced	n=1025	n=229	<0.001^b^
Planning pregnancy	338 (33.0)	41 (18.6)	
Aware of pregnancy	672 (65.6)	177 (80.5)	
Other	15 (1.5)	2 (1)	
Social support (lower score =lower support)	n=1058	n=223	0.707^a^
5-18 points	31 (2.9)	15 (6.7)	
19-24 points	84 (7.9)	11 (4.9)	
25 or more points	943 (89.1)	197 (88.3)	
Tobacco use before pregnancy	n=1059	n=222	<0.001^b^
Daily	134 (12.7)	80 (36.0)	
Not daily	66 (6.2)	34 (15.4)	
Quit before pregnancy	142 (13.4)	28 (12.6)	
Never used	717 (67.7)	80 (36.0)	
Drinking motive: social (lower score= less important)	n=1060	n=226	<0.001^a^
4-10 points	917 (86.4)	125 (55.3)	
11-15 points	135 (12.7)	90 (39.8)	
15-20 points	8 (0.8)	11 (4.9)	
Drinking motive: coping	n=1054	n=224	
4-10 points	1044 (99.1)	199 (88.8)	<0.001^a^
11-15 points	7 (0.7)	21 (9.4)	
15-20 points	3 (0.3)	4 (1.8)	
Drinking motive: enhancement	n=1057	n=226	<0.001^a^
4-10 points)	990 (93.7)	145 (64.2)	
11-15 points	64 (6.1)	70 (31.0)	
15-20 points	3 (0.3)	11 (4.9)	

^a^p-value based on Man Whitney’s *U*-test.

^b^p-value based on chi-square test.

Responses concerning drinking motives differed between the moderate and hazardous drinkers. For all motives (social, coping, and enhancement), the hazardous drinkers had higher sores than the moderate drinkers indicating the motive is stronger (Table 2).

### Alcohol use during pregnancy

Eighty-nine women reported they had consumed alcohol during their pregnancy: 5.5% of the total sample and 6.5% of the women who drank alcohol in the year preceding the pregnancy (Table 3). Two women who abstained from alcohol the year before pregnancy reported drinking during pregnancy. Of the women who drank during pregnancy, 81 (92%) reported drinking at most 1 SD at the time, one women reported drinking 3-4 SD and 6 women did not answer how much they consumed during pregnancy. A majority of the women (63 women, 72%) stated they had been drinking 1-2 times so far during their pregnancy, 22% (19 women) had been drinking 3-6 times and 6 women who were all in pregnancy week 30 or more, had been drinking more often The prevalence rates varied from 0% to 13.9% between the different centers.

**Table 3:** Sociodemographics, drinking, and smoking among the women who drank during their pregnancy and those who abstained

	**Consumers during pregnancy, *n *(%)**	**Abstainers during pregnancy, *n *(%)**	***p*-value**
Age (years)	n=88	n=1213	<0.001^b^
≤ 24	4 (4.5)	132 (10.9)	
25–29	19 (21.6)	403 (33.2)	
30–34	34 (38.6)	441 (36.4)	
35–39	23 (26.1)	202 (16.7)	
≥ 40	8 (9.1)	35 (2.9)	
Education	n=89	n=1192	0.041^b^
Compulsory school	2 (2.2)	23 (1.9)	
Intermediate education	24 (26.9)	464 (38.9)	
University/college education	63 (70.8)	705 (59.1)	
Occupation	n=89	n=1212	0.508^b^
Employed	76 (85.4)	997 (85.1)	
Other	13 (14.6)	215 (14.9)	
City size	n=89	n=1222	<0.001^b^
>200 000 inhabitants	45 (50.6)	385 (31.5)	
<200 000 inhabitants	44 (49.4)	837 (68.5)	
Region	n=89	n=1466	0.018^b^
Norrland1)	21 (23.6)	491 (33.5)	
Svealand2)	29 (32.6)	306 (20.9)	
Götaland3)	39 (43.8)	669 (45.6)	
Drinking habit	n=84	n=1188	<0.001^b^
Strong	9 (10.7)	23 (1.9)	
Medium	23 (27.4)	124 (10.4)	
Weak	52 (61.9)	1041 (87.6)	
Social support	n=87	n=1203	0.035^a^
Low	3 (3.4)	44 (3.7)	
Medium	8 (9.2)	90 (7.5)	
Adequate	76 (87.4)	1069 (88.9)	
Drinking motive: social	n=84	n=1180	0.001^a^
Unimportant	57 (67.9)	970 (82.2)	
Moderately important	25 (29.8)	194 (16.4)	
Important	2 (2.4)	16 (1.4)	
Drinking motive: coping	n=85	n=1171	<0.001^a^
Unimportant	79 (92.9)	1147 (97.8)	
Moderately important	6 (7.1)	20 (1.7)	
Important	0 (0)	6 (0.5)	
Drinking motive: enhancement	n=85	n=1176	0.002^a^
Unimportant	75 (88.2)	1042 (88.6)	
Moderately important	10 (11.8)	121 (10.3)	
Important	0 (0)	13 (1.1)	
Tobacco use before pregnancy	n=87	n=1208	0.871^b^
Daily	15 (17.2)	204 (16.9)	
Not daily	5 (5.7)	95 (7.9)	
Quit before pregnancy	10 (11.5)	161 (13.3)	
Never used	57 (65.5)	748 (61.9)	
Tobacco use during pregnancy	n=88	n=1209	0.046^b^
Daily	4 (4.5)	44 (3.6)	
Not daily	10 (11.4)	55 (4.5)	
Not at all	74 (84.1)	1110 (91.8)	
Frequency of drinking before pregnancy	n=86	n=1213	
Once a month or less	14 (16.3)	491 (40.5)	<0.001^b^
2–4 times a month	55 (64.0)	644 (53.1)	
2–3 times a week or more	17 (19.8)	78 (6.5)	
Usual quantity per occasion before pregnancy	n=86	n=1212	0.649^b^
1 SD	16 (18.6)	198 (16.3)	
2 SD	40 (46.5)	521 (43.0)	
3–4 SD	23 (26.7)	357 (29.5)	
5 or more	7 (8.2)	136 (11.2)	

^a^p-value based on Man Whitney’s *U*-test.

^b^p-value based on chi-square test.

Sociodemographics as well as alcohol consumption preceding the pregnancy differed between the women who abstained from alcohol during the pregnancy and those who continued drinking alcohol. The women who drank during pregnancy were more likely to be older than the women who ceased drinking, have a university or college education, and live in a major city. The strength of the prepregnancy alcohol habit was stronger among those who drank during pregnancy. These women also had a higher frequency of drinking before becoming pregnant. Scores on social, coping and enhancement motives for drinking were higher among the women who drank during pregnancy and they more often used tobacco during pregnancy (Table 3).

### Predictors for drinking during pregnancy

Six predictive factors of alcohol consumption remained in the final logistic regression model; higher age, living in a large city, tobacco use during pregnancy, lower MSSS score, lower SRHI score and higher score on social drinking motives (Table 4). The factors excluded from the model were; education, frequency and quantity of alcohol consumption before pregnancy, frequency of binge drinking before pregnancy, tobacco use before pregnancy, enhancement motives, coping motives and region.

**Table 4:** Logistic regression for drinking during pregnancy

	**Odds ratio**	**Confidence interval (95%)**	***p*-value**
Age			
≤ 24	1	–	
25–29	3.09	0.65-14.63	0.155
30–34	4.54	0.98-20.97	0.053
35–39	8.51	1.80–40.32	0.007
≥40	11.32	1.85-96.15	0.009
City size			
<200 000 inhabitants	1	–	
>200 000 inhabitants	1.69	1.00-2.86	0.048
Tobacco use during pregnancy			
Not at all	1	–	
Not daily	1.26	0.27-5.74	0.768
Daily	3.76	1.57-9.00	0.003
Social support	0.87	0.77-0.98	0.028
Drinking habit	0.86	0.80-0.93	<0.001
Social drinking motives	1.12	1.00-1.26	0.044

The Nagelkerke R square value = 0.150n.

Women who were 40 years or older were more than 11 times more likely and those aged 35–39 years were eight and a half times more likely to drink during pregnancy compared with those aged 24 years or younger. Women visiting antenatal care centers in the large cities were more likely to drink during pregnancy compared with women in smaller cities and rural areas. The women who used tobacco daily during their pregnancy were almost four times more likely to drink during pregnancy than the women who did not use tobacco at all. Higher social support and weak alcohol habit before pregnancy were associated with decreased risk of drinking during pregnancy respectively. Higher score for social drinking motives increased the likelihood of drinking during pregnancy with 12% (Table 4).

## Discussion

This study has sought to investigate alcohol use before and during pregnancy and predictors for drinking during pregnancy in Sweden. Approximately 6% of the women consumed any alcohol during their pregnancy. Older age, living in a large city, using tobacco during pregnancy, lower score for social support, stronger alcohol habit before pregnancy and higher score for social drinking motives were factors found to predict drinking during pregnancy.

We found that 84% of the women consumed alcohol the year preceding pregnancy, with 69% being moderate drinkers, 15% hazardous drinkers and 16% reporting prepregnancy abstinence. Four-fifths of the women with hazardous drinking before the pregnancy continued to drink until they became aware of their pregnancy, suggesting that there is a substantial risk that they consumed alcohol after becoming pregnant but before pregnancy recognition. In a study on pregnancy planning [28] it was reported that only 10% of the women changed their pattern of alcohol consumption during the pregnancy planning period. Since alcohol consumption can harm the fetus in the earliest weeks of pregnancy, even before pregnancy recognition [29], it is of importance to find ways to prevent drinking in early pregnancy.

Abstinence after pregnancy recognition was achieved by almost all women. Most of the women who did not cease drinking during pregnancy reported drinking small amounts and few drinking occasions. These findings are similar to earlier studies conducted at a single antenatal care center in Sweden, showing prevalence rates of about 6% after pregnancy recognition [11,17]. However, these studies used a retrospective questionnaire answered at home after giving birth. Other Swedish studies investigating the prevalence of alcohol consumption during pregnancy reported prevalence rates between 12% and 30% [10,12]. These studies were single-centre studies undertaken in Stockholm, the capital of Sweden, and Uppsala, the fourth largest city in Sweden. These higher prevalence figures are in line with our findings showing that the prevalence of alcohol use during pregnancy is approximately twice as high in the three major cities included in this study compared with smaller cities and rural areas. Population-based data show a similar pattern of higher alcohol consumption in the major cities compared with the rest of Sweden [8]. Since the prevalence rates vary from 0-13.9% in the 30 antenatal care centers included in this study, it is not surprising that the prevalence rates reported in single center studies vary a great deal.

Factors found to predict drinking during pregnancy were higher age, living in a major city, tobacco use during pregnancy, low social support, strong prepregnancy alcohol habits and higher score for social drinking motives. Some of these predictors are consistent with previously identified factors as age and smoking [30]. Since smoking like alcohol is harmful to the foetus it can be of importance to be aware of the association from a preventive point of view. We have not found any international studies that support our finding that a higher share of women in larger cities consumes alcohol during pregnancy compared to women in smaller cities or rural areas. However, this likely echoes the drinking cultures and norms present in the cities and rural areas. The developers of MSSS has found that the scores are correlated with poorer health during pregnancy, contacting antenatal care later and more depressed mood after delivery [25]. Our findings indicate that the instrument could also be useful to identify women with elevated risk for consuming alcohol during pregnancy, although this needs to be further investigated.

Pre-pregnancy habits were also found to predict drinking during pregnancy. Although we have not identified any studies investigating alcohol habits in a pregnant population, pre pregnancy drinking frequency/drinking behavior have been found to be the strongest predictor of drinking during pregnancy in several studies [10,31,32]. Repetition of a behavior in a stable context is required for developing a habit, but the association between frequency of enacting a behavior and habit strength is not fully understood [33]. Some behaviors turn into habits quickly, whereas others may require years of repetition [34]. Research in various domains has shown that habits performed in stable contexts are unlikely to be spontaneously reconsidered. Because habits are triggered automatically in response to contextual cues, some sort of contextual change or disruption might be needed to make behavior-relevant information more salient and influential [35-37]. It seems likely that pregnancy represents such a contextual change, providing a window of opportunity to break habits such as drinking alcohol, as has been suggested in previous research [38]. This preparedness provides an advantage in preventive interventions targeting pregnant women.

The results from this study suggest that the five-question habit instrument SRHI could be used as a screening tool to identify women with risk of drinking after pregnancy recognition although this needs to be further investigated. The instrument has been used with numerous behaviours [39], but not with pregnant women. In current Swedish antenatal care all women are screened for prepregnancy alcohol use with the 10-item AUDIT questionnaire with the aim of finding women with increased risk of continuing drinking during pregnancy. Three out of four women who drank during pregnancy in our study did not have hazardous consumption in the year preceding pregnancy. Although we did not use the full AUDIT instrument, our results suggest that screening for hazardous prepregnancy alcohol intake might not be optimal to identify the women who will drink during pregnancy. This is in line with Magnusson [40], who found that most women who drink during pregnancy did not have AUDIT scores indicating likely alcohol dependence why further research to find more effective screening instruments is vital.

Differences in motives for drinking have been found to predict patterns of alcohol consumption and to be a risk factor for drinking in nonpregnant populations [24,41]. Pregnant women in this study who consumed alcohol during pregnancy were more likely to score higher on social drinking motives for drinking in the year preceding pregnancy. Social motives have previously been associated with moderate alcohol use, coping motives with frequent but not heavier drinking, and enhancement motives with heavy drinking [24,41].

This study has some limitations that need to be considered when interpreting the results. We used self-reporting of alcohol consumption, which is a source of uncertainty because the responses might be influenced by social desirability, a bias that tends to be important when the questions deal with socially desirable (or undesirable) attitudes and behaviors [42]. However, when assessment situations are structured to minimize bias self-reports show adequate reliability and validity [43]. To reduce the risk of social desirability bias in the present study, the respondents were guaranteed anonymity, which has been shown to reduce bias in self-reports of sensitive behaviors [44].

We sought to achieve a sample which was representative of the distribution of pregnant women in Sweden. The invitations went through coordinating midwives who are in charge of a number of antenatal care centers in a region. Some of the coordinating midwives passed the invitation on to all centers in the region while others asked only a few centers. The research team was then contacted by or given contact information to centers willing to participate. Unfortunately, response rates for the invited centers are not available. It was not possible to obtain the precise number and proportion of pregnant women for all location/city size combinations. Thus, the study population does not entirely reflect the pregnant population in Sweden. Since the prevalence of drinking during pregnancy varied between large and smaller cities as well as between the different regions, it is possible that the reported prevalence rate is not representative of the whole population. On the other hand, our study adds information about differences across the country that has not been shown earlier.

This study required active participation by the midwives at the antenatal care centers to give the questionnaires to the pregnant women. We do not have full information on the extent to which midwives neglected to give out the questionnaire to some women, e.g. due to heavy workload. However, based on our informal assessment of several antenatal care centers, this type of omission was uncommon. Furthermore, this factor is unlikely to have biased the results in any specific direction.

Another potential limitation of this study is the risk of selection bias. This might lead to over- or underestimation of the prevalence of drinking during pregnancy differs between responders and non-responders. The drop-out analysis showed that the non-responders were younger than the responders, making overestimation more likely than underestimation. Further, the questionnaire was available only in Swedish, which meant that women who did not understand Swedish were excluded from the study. This might have affected the prevalence rate since drinking cultures and patterns of consumption vary between different cultures and locations.

A strength in our study is that we achieved a low dropout rate, which can be partially attributed to our efforts to establish good relationships with all participating antenatal care centers and to generate interest in the study. The first author visited nearly all centers in person to inform about the study and discuss various issues about the study procedure. To avoid burdening the midwives, data collection was restricted to a 4-week period at each center. All participating centers received a report with their unique data. Compared with previous Swedish studies conducted at single centers, our multicenter approach is an advantage because it increases the generalizability of the findings to the entire population. Another strength is the assessment of several predictors for drinking during pregnancy that have not previously been examined in a Swedish setting.

## Conclusions

This study revealed that the prevalence of drinking alcohol during pregnancy is relatively low in Sweden, as 5.5% continued to consume alcohol after pregnancy recognition. However, 84% of the women reported drinking in the year preceding pregnancy and most of these women continued drinking until pregnancy recognition, which means that they might have consumed alcohol in their early pregnancy. Higher age, living in a major city, using tobacco during pregnancy, low social support, stronger drinking habit before pregnancy and higher score for social drinking motives were found to predict drinking during pregnancy. These factors should be addressed when planning interventions to reduce alcohol consumption during pregnancy.

## Competing interests

The authors declare that they have no competing interests.

## Authors’ contributions

JS and PN conceived and designed the study and drafted the manuscript. JS coordinated data collection. SA and EHN participated in study design and interpretation of the findings. KÅ and JS performed the statistics. SA, EHN and KÅ revised the manuscript. All authors read and approved the final manuscript.

## Pre-publication history

The pre-publication history for this paper can be accessed here:

http://www.biomedcentral.com/1471-2458/13/780/prepub
